# Brazilian intensivists: exhausted, but (still) happy with their choice?

**DOI:** 10.5935/0103-507X.20160047

**Published:** 2016

**Authors:** Fernando Godinho Zampieri

**Affiliations:** 1 Instituto de Pesquisa, Hospital do Coração - São Paulo (SP), Brazil.; 2 Intensive Care Unit, Hospital Alemão Oswaldo Cruz - São Paulo (SP), Brazil.

**Figure 1:**
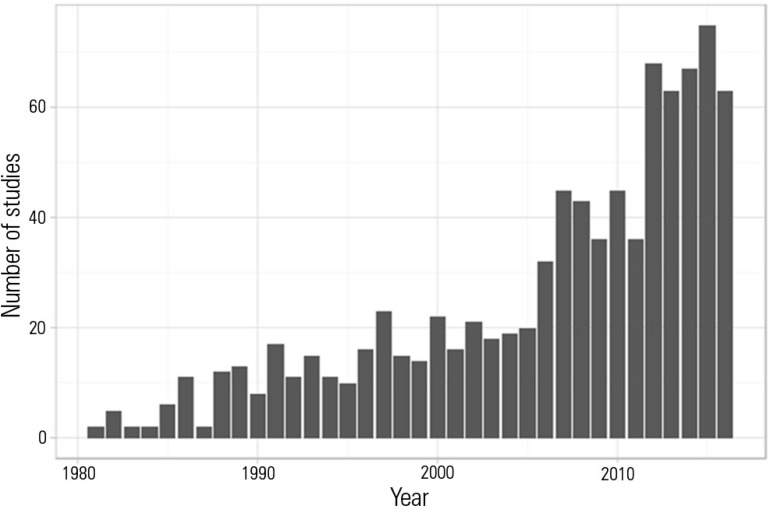
Number of articles published by year indexed before the end of July 2016 that included the term "burnout" in the title or in the abstract. Despite the increase in the last five years, the number of articles on the subject is still surprisingly small.

Burnout syndrome has gained increasing attention since the term was coined by Freudenberger in 1974.^(1)^ It is amazing that the first systematic description of occupational physical or mental burnout was provided more than two centuries after the modern contextualization of work.^(2)^ It is even more astonishing that more than 40 years have passed without any studies assessing the actual implications of burnout syndrome on the work dynamics in healthcare and the outcomes on patient care. Despite a significant increase in the number of studies on this subject that have been published in the last five years (Figure 1), the number is still very small compared to studies of other occupational diseases (not exceeding approximately 70 articles on the topic per year).

In this edition of the *Revista Brasileira de Terapia Intensiva*, Tironi et al.^(3)^ report the results of a systematic questionnaire completed by 180 Brazilian intensive care physicians from five state capitals. The prevalence of symptoms of emotional burnout, depersonalization, and inefficiency was evaluated using the abovementioned previously validated systematic questionnaire. The authors were careful to make their sample representative of the overall population by including 60 intensive care units in large Brazilian capitals; however, the return rate of the questionnaires was considerably lower than expected (despite the abovementioned refusal to participate by some centers). Thus, before interpreting the study results, we must emphasize that they refer to professionals willing to complete the questionnaire in units that agreed to participate in the study. Thus, it is possible that a larger sample would lead to even more alarming results. The authors found a burnout prevalence of greater than 60%, when considering burnout as the presence of at least one of the domains involved.

The results of Tironi^(3)^ must be interpreted in conjunction with the results of other international initiatives and with the data on professional and personal satisfaction among intensivists published by Nassar, also in *Revista Brasileira de Terapia Intensiva*.^(4)^ The Brazilian intensivist is young, often well paid, but subject to a high weekly workload. As with other doctors in other parts of the world, emotional exhaustion is the most frequently reported component of burnout syndrome.^(5)^ However, Brazilian intensivists seem to suffer from burnout more frequently than their Portuguese colleagues.^(6)^

We know little about the consequences of burnout. The direct relationship between burnout and worse care performance, although plausible, has yet to be demonstrated in practice. Some data suggest that patient satisfaction is lower when the assistant physician suffers from burnout; however, the link between the presence of the syndrome and worse outcomes still needs to be clearly demonstrated.^(7)^ There is, however, indirect evidence. Emergency physicians with burnout report providing sub-optimal care on a greater number of occasions.^(8)^ In an important study, Welp et al. demonstrated that emotional exhaustion in intensive care physicians and nurses negatively influences interprofessional care provision and patient safety culture in intensive care units, thereby stressing the importance of performing surveillance, recognition, and treatment of burnout syndrome.^(9)^

A shortage of intensive care physicians exists in Brazil and worldwide.^(10)^ In addition to the direct consequences to the care provided, the high prevalence of burnout in intensivists can lead to the migration of professionals from intensive care to other healthcare areas, especially in settings where many physicians work in other specialties concomitantly to intensive care medicine, as is the case of Brazil.^(4)^ In a questionnaire applied to healthcare professionals involved in the care of cancer patients, where emotional exhaustion rates similar to those of the study by Tironi^(3)^ were obtained (53.3% *versus* 50.6%), approximately one-third of the respondents reported an intention to seek a different job.^(11)^ It is unclear whether an association exists between burnout and a tendency to change jobs among Brazilian intensivists, as reasonable levels of satisfaction with their professional choice were reported.^(4)^ The scenario, however, does not seem encouraging. As reported by Tironi,^(3)^ the bureaucratic burden and the lack of time for addressing the emotional needs of the patient, along with the inevitability of facing family conflicts, are frequent complaints among Brazilian intensivists. As the first complaint is unable to assess the value of the second and both are related to the third, a system is created in which the user, rather than being empowered, becomes adrift.^(12)^ It should be noted that I refer to the dysfunctional bureaucracy, as described by Merton, and not the development of essential care protocols aimed at helping the physician and improving outcomes.^(13)^ The next years will determine whether satisfaction of Brazilian intensivists with their profession persists or if burnout will prevail.
